# Computational analysis of size, shape and structure of insect wings

**DOI:** 10.1242/bio.040774

**Published:** 2019-10-15

**Authors:** Mary K. Salcedo, Jordan Hoffmann, Seth Donoughe, L. Mahadevan

**Affiliations:** 1 Department of Organismic and Evolutionary Biology, Harvard University, Cambridge, MA 02138, USA; 2 School of Engineering and Applied Sciences, Harvard University, Cambridge, MA 02138, USA; 3 Department of Molecular Genetics and Cell Biology, University of Chicago, Chicago, IL 60637, USA; 4 Department of Physics, Harvard University, Cambridge, MA 02138, USA; 5 Kavli Institute for Nanobio Science and Technology, Harvard University, Cambridge, MA 02138, USA

**Keywords:** Image quantification, Wing morphology, Phenotyping

## Abstract

The size, shape and structure of insect wings are intimately linked to their ability to fly. However, there are few systematic studies of the variability of the natural patterns in wing morphology across insects. We have assembled a dataset of 789 insect wings with representatives from 25 families and performed a comprehensive computational analysis of their morphology using topological and geometric notions in terms of (i) wing size and contour shape, (ii) vein topology, and (iii) shape and distribution of wing membrane domains. These morphospaces are complementary to existing methods for quantitatively characterizing wing morphology and are likely to be useful for investigating wing function and evolution. This Methods and Techniques paper is accompanied by a set of computational tools for open use.

This article has an associated First Person interview with the first author of the paper.

## Abstract (precis)

**Summary:** We provide a set of simple quantitative measures to compare morphological variation in size, shape, and structure of insect wings across species, families and orders.

## INTRODUCTION

**Fig. 1.**
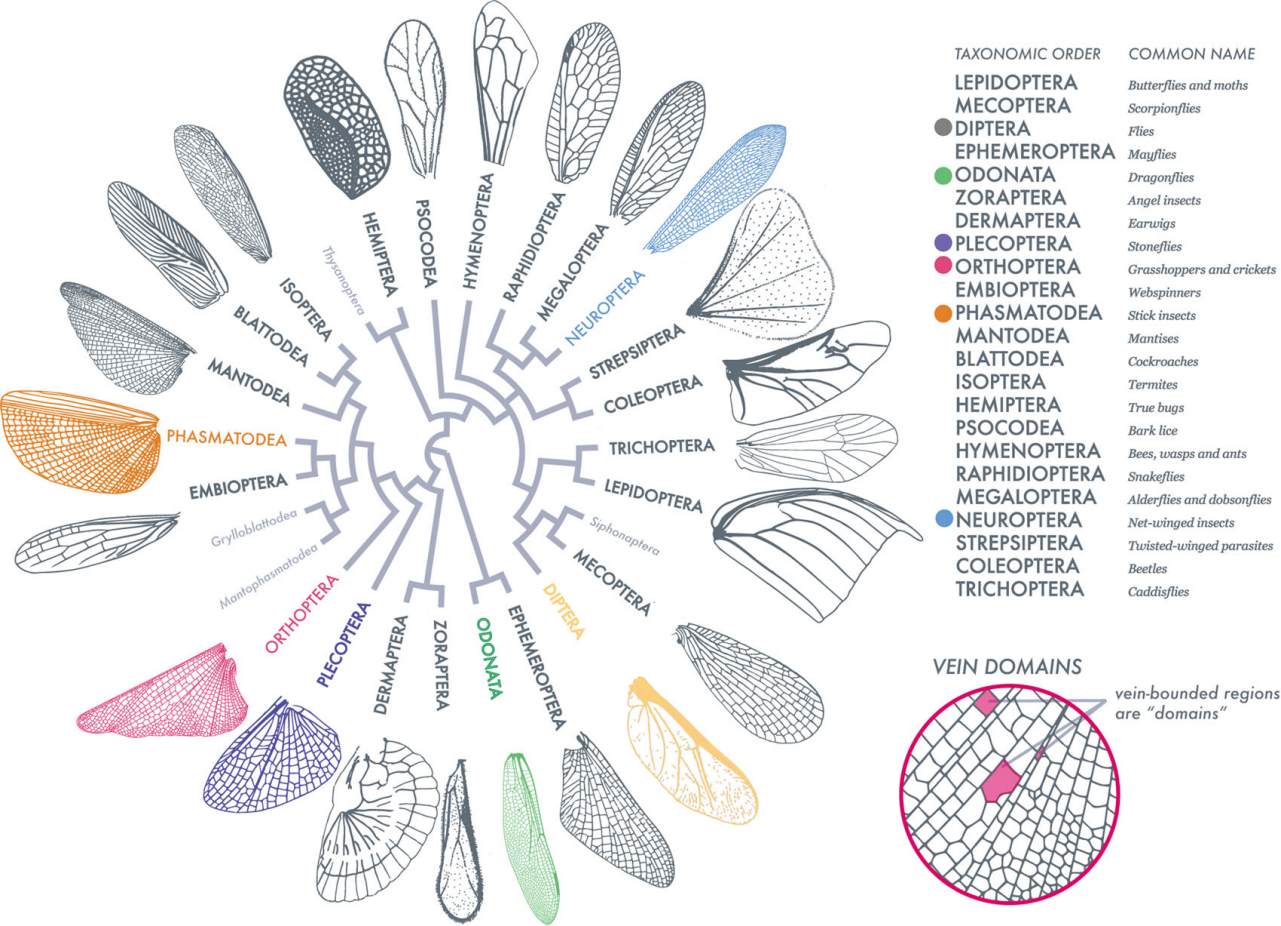
**Insect wing venation patterns, wing contour and shapes of vein-bounded domains.** Adapted from Misof et al. (2014), this order-level representation of insect wing venation patterns, shapes and vein domains (as defined in the figure on the right) exhibits the range and diversity of our sampling. Insect orders labeled in light gray are not sampled or characterized as wingless (full species list in Fig. 5A). Wing scale is removed to compare venation, however wing length ranges an order of magnitude. For example, the Odonate wing (green) is 52 mm in length, compared to the 3 mm in length Dipteran wing (yellow). Six orders (bulleted in color) are highlighted throughout this analysis.

There are more than one million described insect species. Most are capable of flight with wings that show a range of hierarchies of complexity (Fig. 1). Wings vary in size, venation, stiffness and flexibility (Combes and Daniel, 2003), and flight behaviors (Betts and Wootton, 1988; Wootton, 1992, 1981), while being subject to strong selective pressures such as ecological niche specialization (DeVries et al., 2010; Johansson et al., 2009; Suárez-Tovar and Sarmiento, 2016).

From a physical perspective, insect wings are slender quasi two-dimensional membranes criss-crossed by a network of tubular veins. The patterns formed by veins can partition some wings into just a few domains (vein-bounded regions of wing membrane) and others into many thousands. The venation network allows for fluid and nutrient transport across the structure while providing a mechanical skeleton that maintains wing stiffness (Wootton, 1992; Brodsky, 1994; Jongerius and Lentink, 2010; Rees, 1975). The outer contour of the wing forms a complex continuous path; it also varies dramatically across insects. With vast differences in venation density and branching (e.g. comparing a Dipteran wing versus an Odonate wing), comparing vein geometries between insect taxonomic orders is not trivial.

Measuring geometric and topological complexity across diverse taxa has been a longstanding issue and presents several challenges: (1) compiling a sufficient dataset of wings, (2) accurately segmenting images of wings and, lastly, (3) normalizing wing shapes. While phylogenetic analyses and geometric morphometrics have been deployed to understand the variation of and selection pressures on wing morphology (Wootton, 1992; Johansson et al., 2009; Suárez-Tovar and Sarmiento, 2016; Nijhout, 2001; Debat et al., 2009; MacLeod, 2007; Pélabon et al., 2006; Parchem et al., 2007), their scope has been limited to a few species or orders at most. In part this is due to the necessary homologous information needed to complete these analyses such as specific markers on venation (Suárez-Tovar and Sarmiento, 2016). In order to quantify wing geometries and complexity between orders, a coarse-grain method of analyzing wing morphology is needed.

Ellington (1984) performed one of the first comparative efforts to parameterize insect wing shape. He characterized ‘gross’ (mass, body length, wing length, wing area and wing mass) and ‘shape’ wing parameters to quantify mechanical aspects of flight for five insect orders. Expanding on comparative wing analysis, Combes and Daniel (2003) quantified flexural stiffness and venation patterns in 16 species spanning six orders, but focused on forewings. They characterized scaling relationships and patterns of wing flexibility along the spanwise and chordwise wing axes. While these works uncovered commonalities in wing morphology, they were limited by their sample size and the detail with which they quantified wing traits.

Here we complement these studies by using a set of simple quantitative measures to compare morphological variation in size, shape, and structure of insect wings across species, families and orders. We start by assembling 789 wings drawn from 24 taxonomic orders, sampling representatives from nearly every extant insect order. We then deploy geometrical and topological methods on these structures, to normalize wing size, quantify shape, characterize venation complexity and break down the distribution of domain sizes and shapes. We highlight six analyzed orders from our dataset (Fig. 1) with varying levels of wing complexity. Our dataset and analysis will, we hope, serve as a first step in dissecting wing complexity and be used in functional and phylogenetic approaches in order to test hypotheses about wing evolution and physiology.

## RESULTS

**Fig. 2.**
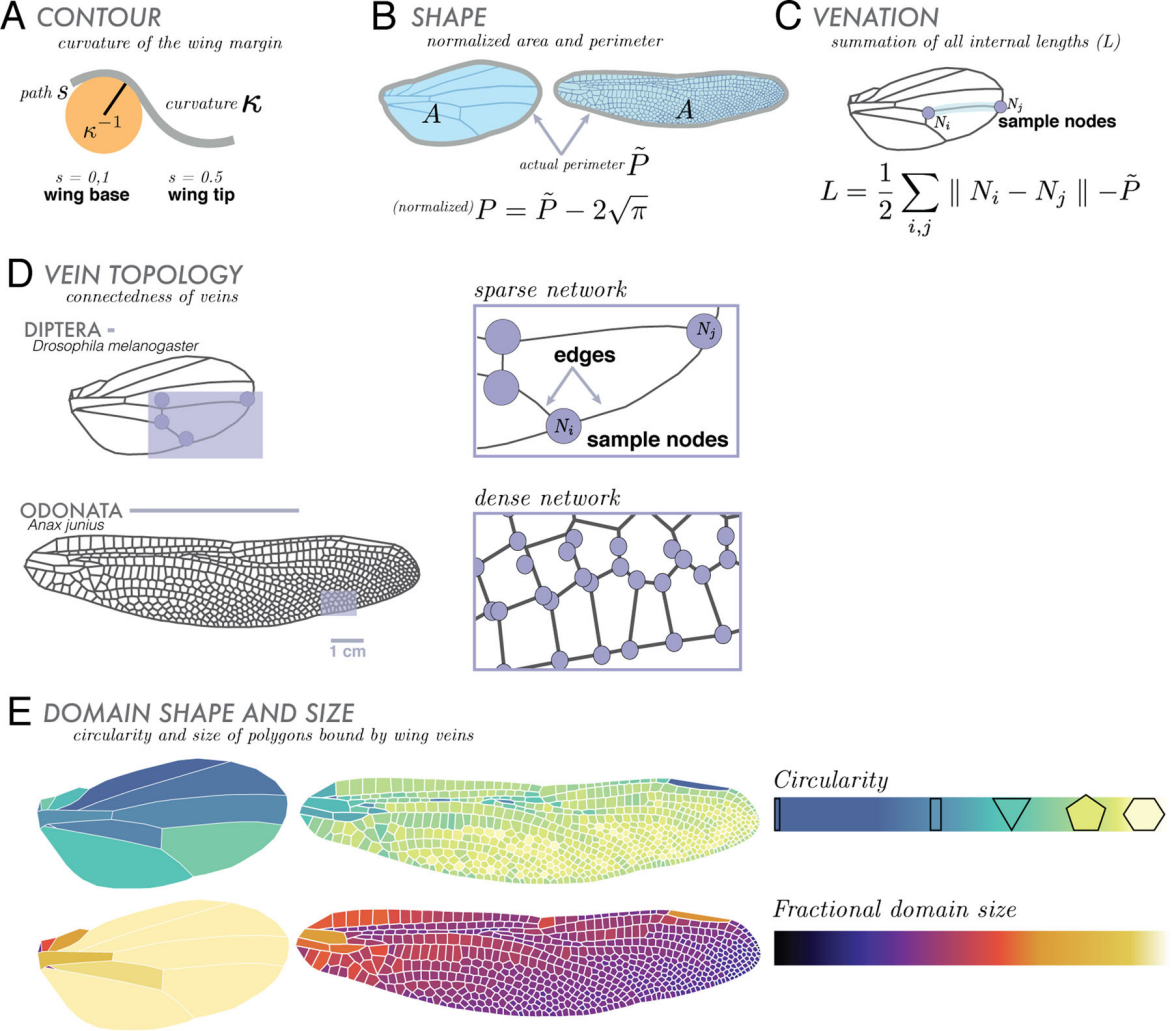
**Overview of morphometric approaches.** We focus on broad comparative geometric and topological components, illustrated here using Diptera (*Drosophila melanogaster*) and Odonata (*Anax junius*) wings as examples. For geometric features, we analyze curvature, shape and area, and internal venation. (A) Contour, *κ*, is given by the radius of curvature or *κ*^−1^ (where *s* is arc length along the wing). (B) Shape: all wings are normalized to have an area equal to that of a circle with an area of unity (removing absolute size effects). Wing shape is characterized by its scaled perimeter, *P*, where *P˜* is the actual perimeter of the wing. (C) Venation is treated as a network, and quantified in terms of the sum of its total internal length, *L*, where *N*_*i*_ and *N*_*j*_ are representative nodes. (D) We continue analyzing venation using topological measures the wing is represented as a network of vein junctions (nodes) and the lengths of vein between them (edges). Lastly, we observe the geometries and distributions of vein domains. (E) Domains, vein-bounded regions, are characterized by their circularity (shape relative to that of a circle) and fractional domain size (domain area relative to area of entire wing). These internal shapes can range from tenths of a millimeter to several centimeters.

Using our compiled geometrical and topological datasets, we calculated the basic geometric features of a wing: contour-curvature (Fig. 2A), scaled wing size and shape (Fig. 2B), internal venation length (Fig. 2C) and connectedness in venation topology (Fig. 2D). Since absolute wing size in insects is roughly correlated with body size (Grimaldi and Engel, 2005), we do not consider size directly. Each segmented wing is normalized by comparing its shape and wing contour to the shape and curvature of a circle. We also studied the distributions of geometric shapes formed by veins within each wing. These domains – regions bound by veins – are assessed by their shapes in terms of two simple statistical measures (Fig. 2E): (1) circularity, the ratio of domain perimeter to the circumference of a circle with the same area and (2) fractional area, the ratio of each individual domain area to the area of the entire wing. Together, these metrics serve as complementary features for quantifying the range of morphological characteristics of insect wings.

### Wing contour-curvature, scaled perimeter and internal venation length

**Fig. 3.**
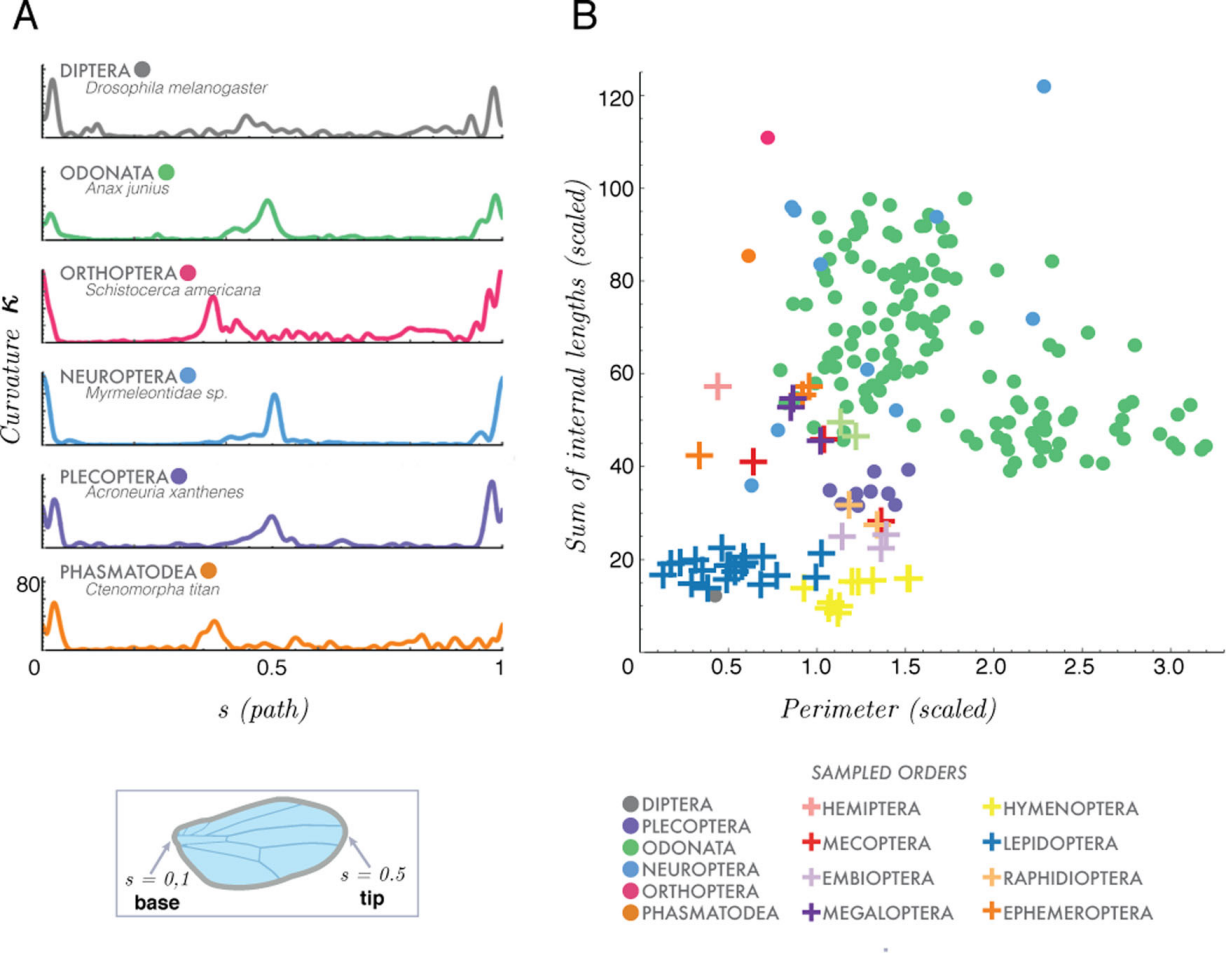
**Wing shape, contour and internal venation.** (A) Comparison of three geometric traits (of all sampled orders) where contour is defined using a scaled curvature (*κ*) (scaled by total perimeter *P*) as a function of arc length (or path), *s*. Curvature is plotted along path *s* where *s*=0 at the wing base and *s*=0.5 at the wing tip. Contour for six orders is represented. (B) Our second trait characterizes venation density of 298 wings, where the total sum of all internal vein lengths, *L* (scaled by the perimeter *P*), is plotted as a function of our third trait, normalized perimeter *P* (scaled by the square root of the area of the wing, see text). Species (per insect order) are represented by either circles or crosses.

Our first morphospace characterizes wing shape complexity treated in terms of its boundary curvature *κ*(*s*) as a function of the arc-length distance from the wing hinge (Fig. 2A). In Fig. 3A, we show a plot of absolute wing curvature (non-negative) varying from wing base (*s*=0 and *s*=1) to wing tip (*s*=0.5) for representative wings from six orders (top to bottom): Diptera, Plecoptera, Odonata, Neuroptera, Orthoptera and Phasmatodea.

Our second morphospace describes the relationship between the density of interior venation and wing contour. Here we explore two geometric features: scaled wing perimeter *P* (Fig. 2B), and the scaled interior venation network, where *L* is the summation of all lengths of interior vein connectivity within the wing [which excludes perimeter (Fig. 2C)]. Both of *P* and *L* are normalized by the square root of the wing area in order to isolate total vein length from overall wing size (Fig. 2B). Then, we define the normalized perimeter,(1)
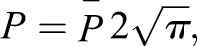


so that a circle with unit area would have *P*=0. We plot the scaled wing contour length *L* against venation contour *P*, Fig. 3B, noting that a wing at the origin (0, 0) corresponds to a circular wing without any internal venation. We see that wings with dense venation (e.g. locust, Orthoptera) occupy the upper middle/right sections of the graph and wings with sparse venation (e.g. fruit fly, Diptera) occupy the lower/left regions of the morphospace.

### Vein topology of a wing

**Fig. 4.**
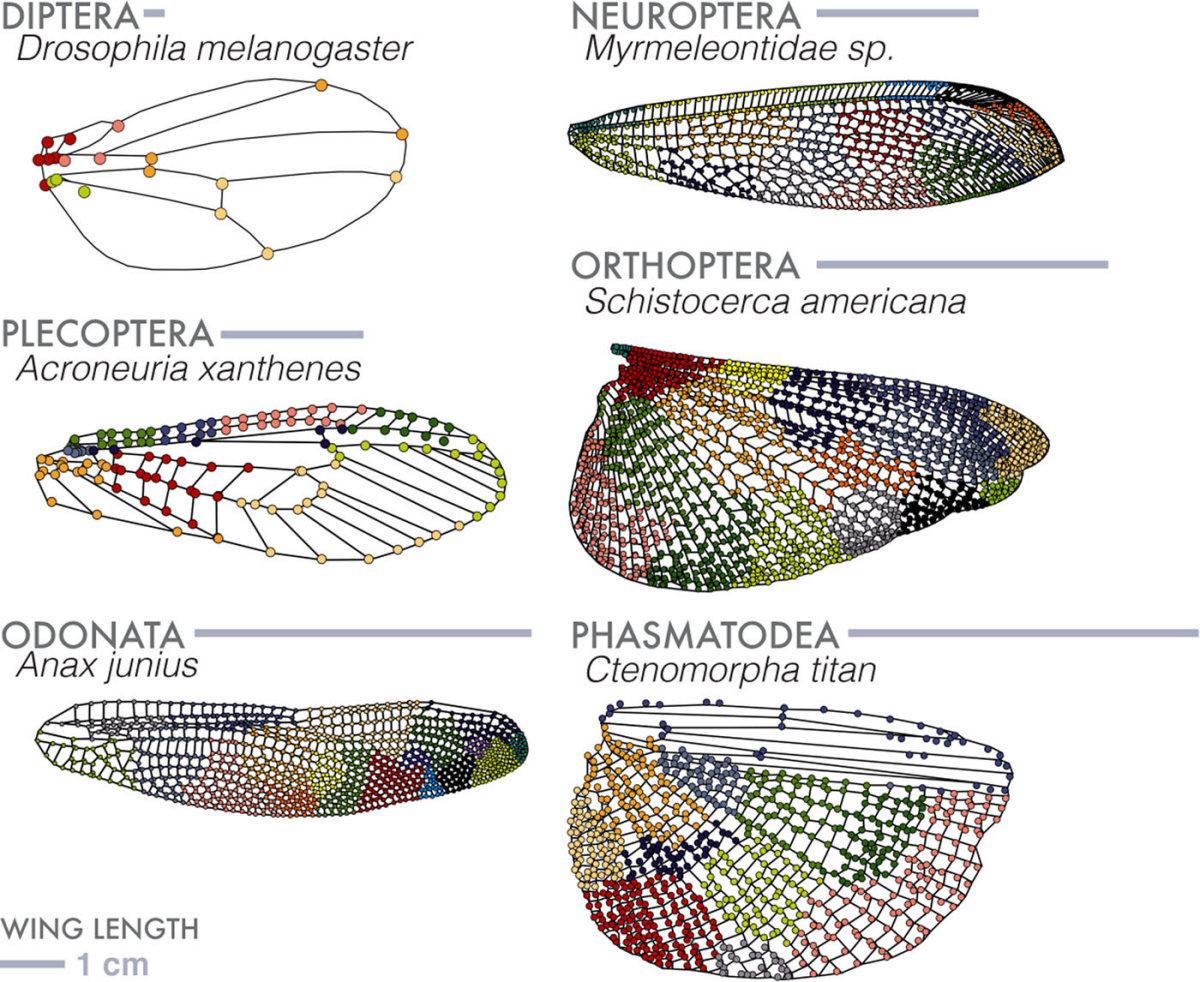
**Topologies of wing venation networks.** A wing can be considered as a network made up of vein junctions (nodes) and the lengths between them (edges). Using segmented wing images, where each 2D component of the wing is mapped out, we characterize a network using a common community detection algorithm, maximum modularity. Here we show a sampling of wing types and their resultant patterning of clusters, or communities. These communities describe node densities where vein junctions of high relatedness are grouped, based on proximity. Colors of communities represent relatedness.

Wing venation forms a physical network with the intersections of veins as nodes (Fig. 4). We used tools from network analysis (Lasser and Katifori, 2017; Dirnberger et al., 2015) to cluster the network into communities quantifying a third major trait of a wing: a topological measure of the complexity of venation patterns. An insect wing can contain a venation hierarchy of varying diameters and lengths, where large longitudinal veins can branch and be connected to smaller cross veins. Though not visible in every venation pattern, hierarchies can often be identified through simple network analysis.

We started by building an unweighted symmetric adjacency matrix, *A*, where every node_*ij*_=node_*ji*_ (see Fig. 2D). To partition a wing network into clusters or communities (Morrison and Mahadevan, 2012), we used the maximum modularity measure, which compares a given network to a randomly generated network and is maximized by a partition factor (Newman, 2016). This allowed us to determine the number and size of clusters, each of which indicates a higher density of internal connections within a group of nodes relative to connections across clusters. In Fig. 4, we show range of venation patterns seen in wings. For wings with sparse venation there are few clusters, e.g. Diptera and Hymenoptera, whereas those with dense venation shows many clusters, e.g. Orthoptera and Odonata.

### Domain circularity and distribution within a wing

**Fig. 5.**
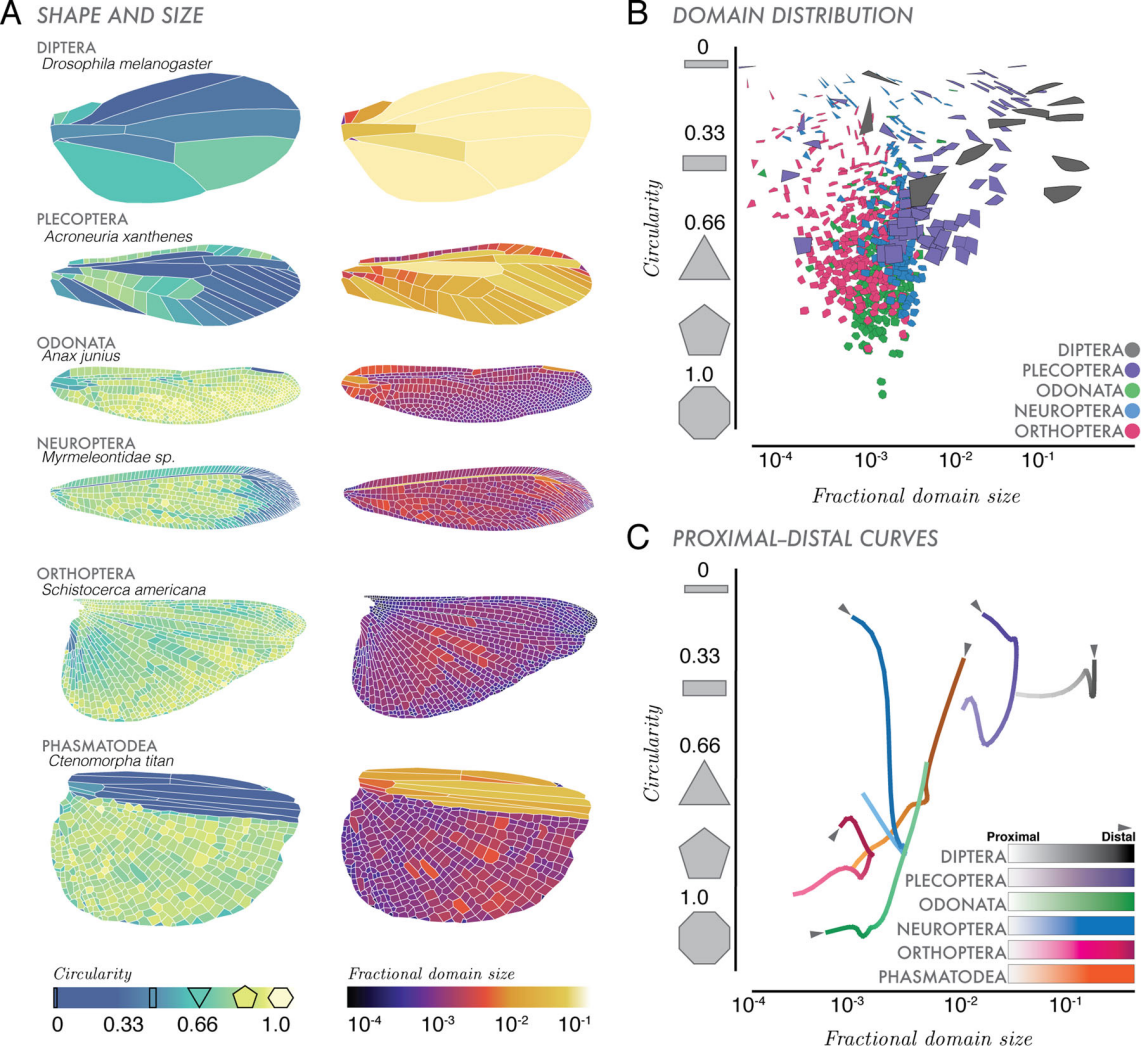
**Domain shapes, sizes and distributions.** (A) Circularity and fractional area characterize the distribution of polygonal shapes that make up the vein-bounded domains within wings. (B) Domain distributions of all the domains within a wing for five representative wings. Color distinguishes domains for each of the species represented (i.e. dark gray domains are from Diptera, *D. melanogaster*). (C) Along the proximal (wing base) to distal (wing tip) (P–D) axis across a wing, we show an average distribution of domain size and area, where fading color (lighter=wing base, darker=wing tip), indicates span of the wing for six insect orders.

In addition to whole-wing topological and geometric features, we also considered fine-grain features. We show in Fig. 5 that domain circularity and fractional area vary within a wing, providing a geometrically minimal description of the internal structure of a wing. Wings with sparse venation tend to have more rectangular domains, while wings with dense venation tend to have higher numbers of more circular domains. For example, in Fig. 5A Dipteran wings have larger, more rectangular domains (dark blue), while Odonate wings are made up of smaller, rounder domains.

Extrapolating on the domains in Fig. 5A, in Fig. 5B, we plot domain distributions from representatives of five insect orders with varying internal complexities of domain shape and venation. For example, all shapes represented in gray are domains found within our Dipteran wing in Fig. 5A. This morphospace quantifies domain shapes (circularity), their distribution in a wing, and how much area they occupy within a wing (fractional area). Within this space, domains at (10^−4^, 1) are small and circular while domains at (10^−1^, 0) are large and rectangular. This morphospace describes the discrete geometries of domains that make up a wing, without respect to where they are located within a wing.

Building on the recent work of Hoffmann et al. (2018), we plotted domain shapes versus how they vary in space across the span of a wing. In Fig. 5C, we consider the proximal to distal axis (P–D axis, wing base to tip) of the wing (similar to wingspan). This axis is divided into *N*=25 rectangular bins, where each bin encompasses all domains across that chord (distance from leading edge and trailing edge of the wing). Following the method of Hoffmann et al. (2018), we then computed the area-weighted mean area and circularity of all domains within each bin. Then we applied a set of normalized coordinates on this P–D axis through the computed domain area-circularity space, which was smoothed with a Gaussian of width 2/25 and rescaled by the wing's perimeter. The resultant J-shaped curves represent the entire distribution of domains across the span of a wing. Similarly to the domain distributions in Fig. 5B, portions of the P–D curves located near (10^−4^, 1) describe regions of the wing that are dominated by small circular domains while portions of curves near (10^−1^, 0) contain domains are characterized by larger, rectangular domains.

For approximately 468 Odonate wings (Fig. 5B,C, green), domains near the wing base (faded green) tend to be rectangular, taking up a larger fractional area of the entire wing. Near the distal end (light green), domains are more circular, taking up less area. A Plecopteran wing (stonefly, purple curve), has an opposite domain distribution: at the wing tip, there are large domains (increased fractional area) that are rectangular (dark purple). Similarly, Neuropteran wings have more elongate and rectangular domains towards the wing tip. Some domains makeup over 10% the total area of the wing (i.e. Diptera), while the smallest account for only 1/10,000 of the entire area (i.e. Odonata) of an insect wing. Our P–D curve morphospace (Fig. 5C) categorizes the average spatial geometries of domains across the wingspan.

## DISCUSSION

Characterizing wing complexity for an insect species requires an accurately segmented wing, homologous landmarks between species (for phylogenetic comparison), and large sample sizes (Johansson et al., 2009; Suárez-Tovar and Sarmiento, 2016). Here, we have compiled a large dataset of wings (both forewings and hindwings) from across the insect phylogeny, quantifying wing complexity using simple topological and geometric features. Using this dataset, we segmented wings with a recently developed technique, (Hoffmann et al., 2018) creating a set of simple morphospaces. These spaces normalize wing shape allowing comparison between highly complex wings (e.g. Odonata) and simpler wings (e.g. Diptera).

Contour-curvature and internal venation, the morphospaces of Fig. 3A and B, quantify the complexity of wing shape (curvature) and venation (sparse or dense). Wings with elongated structures in the forewing or hindwing have distinct contour curvature peaks that serve to identify differences between wing shape. Comparing curvature plots, between species and at the order level, could give insight into wing shape evolution on a broad scale.

The second morphospace (Fig. 3B) quantifies the sum of internal venation (scaled by the perimeter) versus the perimeter (scaled by the square root of the wing area). This space measures internal complexity; wings with sparse venation and circular shapes are found near (0, 0) and wings of dense venation and elongated shapes are found in the upper right of this morphospace. Increased venation density may indicate variable flexural stiffness and thick wing membrane Combes and Daniel (2003). Note that it remains unclear how stiffness varies across the span of a wing and that their measurements exclude the outer 30% of the wing. From their study, certain dragonflies (Odonata, dense venation), moths (Lepidoptera, sparse venation), flies (Diptera, sparse) and bees (Hymenoptera, sparse) have higher flexural stiffnesses than that of certain damselflies (Odonata, dense), craneflies (Diptera, sparse) and lacewings (Neuroptera, intermediate to dense), indicating that variable vein and membrane thickness could play a role in wing stiffness (Combes and Daniel, 2003).

Our third morphospace quantifies the venation pattern as a network, using node density (how closely a group of vein junctions are related) to extract hierarchies within the venation topology. We deployed a suite of network analysis tools on the diverse set of insect wing venation patterns using weighted (Morrison and Mahadevan, 2012) and unweighted analysis (Newman, 2006). Overall, we found the resulting clusters of the wing networks difficult to interpret. This is due in part to how static networks can be interpreted. Once clusters are formed, the information gleaned from them is due to changing dynamics between the groupings. While our clustering of vein connections yields information about the density of venation (dense=many clusters), there needs to be a higher input of wing specific information. With the given networks, further experiments could be performed by changing the weighting parameter between nodes (vein junctions) to have a physical metric. There is a necessity to interpret those groupings using mechanical information (such as stiffness, wing thickness), evolutionary information (key landmarks across species or genus) or with changing structure (potential damage), all important questions that might be studied in the future.

Quantifying wing venation topology to build a parametric model and test bending modes has been deployed on blowfly and cicada wings (orders Diptera and Hemiptera, respectively) suggesting a promising avenue for 3D modeling of insect wings (Mengesha et al., 2009; Sun et al., 2014). Similarly, an analysis using morphological correlation networks, which indicated leaf mimicry in noctuid moths, could be described as a coupling and decoupling of known color patterns (Suzuki, 2013). While our approach utilizes large sample sizes and well-segmented data, more parameters applied to network analysis (i.e. length between nodes, material strength of lengths between nodes, fluidic properties between nodes), are needed. The grouping of nodes within our clustering analysis does not provide concrete conclusions about venation hierarchy. However, in other studies utilizing topological parametric analysis and optimization, the additive use of material properties allowed for finite element modeling (Mengesha et al., 2009; Sun et al., 2014). By providing the adjacency matrices for our wing networks, our hope is that others can use approaches from geometric morphometrics to understand the evolution of wing venation patterns, while also informing modeling efforts for wing flexibility (Jongerius and Lentink, 2010; Mengesha et al., 2009; Sun et al., 2014; Rajabi et al., 2016).

Lastly, we quantified internal complexity of the wing in terms of vein-bounded domains. Domains (Fig. 5A–C), represent ‘building blocks’ that make up a wing. Comparing domain circularity and fractional area in three ways, wing morphology follows a general pattern: sparse venation indicates more rectangular domains with larger fractional areas, and densely venated wings tend to have smaller more circular domains. Wings with high venation density (i.e. high numbers of cross veins) and thus higher numbers of domains (especially towards the trailing edge), are more likely to reduce wing tearing that might occur throughout its lifespan (Dirks and Taylor, 2012; Rajabi et al., 2015). Generally, at the base and leading edge of a wing, vein diameters are larger, and can indicate higher density of wing material and thus higher wing stiffness. Within Odonata, a taxonomic order with large wings and numerous domains (Hoffmann et al., 2018), there is an asymmetry wherein the leading edge has few domains, and the trailing edge is populated with hundreds. An asymmetry regarding domain number and size across the wing span could be beneficial; from a structural integrity perspective, asymmetry provides fracture toughness (Dirks and Taylor, 2012). Since cross-veins effectively transfer tensile stresses to neighboring wing domains (Rajabi et al., 2015), wings with higher numbers of small domains in the trailing edge could reduce damage propagation (Dirks and Taylor, 2012). While not applicable across all orders (i.e. Plecopteran wing in Fig. 5A–C), this asymmetry in domain size and number could indicate flight behaviors where, for example, aggressive predatory flight (i.e. Odonata) necessitates wings that can incur significant damage without affecting function.

Our study provides a broad comprehensive dataset of insect wings and several simple metrics to quantify wing complexity across the phylogeny. Further, entomological texts and published literature contain many more insect wing images and detailed morphological data than we were able to sample. Resources such as the Biodiversity Heritage Library (BHL) provide digitized natural history texts, scanned as large-scale collaborative efforts across universities and libraries. Future work could explore mining BHL for applicable metadata, morphological data and wing drawings, utilizing a rich natural history dataset. This is but a first step in quantifying the morphospaces and the simple morphometric approaches we have outlined only scratch the surface in addressing the origins and functional consequences of insect wings.

## MATERIALS AND METHODS

### Image collection

Our dataset comprises 789 wing images with representatives from 24 out of 30 recognized insect orders (Fig. 1) and comes from a large number of different sources, including original micrographs (hand-caught and donated specimens), a collection of scans from entomological literature (1840s–1930s) sourced at the Ernst Mayr Library, Harvard University (Cambridge, MA, USA) and online through the Biodiversity Heritage Library.

Original micrographs of actual wings were taken using a CanonScan 9000F Mark II and then processed in Fiji/ImageJ using the stitching and extended depth of focus tools. Published wing images where either taken (1) from online resources (Biodiversity Heritage Library) or (2) from books at the Ernst Mayr Library, Cambridge, MA, USA. For images taken from online resources, images were contrast-adjusted with Adobe Photoshop when needed. For images taken from textbooks, images were scanned again with the CanonScan. The supplemental material of Hoffmann et al. (2018) shows an extensive analysis comparing insect wing drawings to micrographs. The authors show that these drawings are true-to-form and accurately capture the geometries in which we are interested.

While we sampled broadly, our data were limited to those insect wings with low pigmentation, or accurate drawings. Wing images from dense entomological texts can have beautifully accurate wing drawings, but not always the morphological information (i.e. size). Since we normalized for wing size, we sampled hundreds of wings to gain insight on geometric complexity of the wings.

Entomological texts were chosen based on the quality and diversity of insects described and how well wing shape, venation, and morphological data were represented, chosen to maximize diversity at the order level. By incorporating newly available data (Hoffmann et al., 2018), we obtained taxonomic coverage enriched for Odonata, a group with particularly complex wings. Insects are referred to by their common order-level names.

Table 1 lists species in the Fig. 1 phylogeny. Insects hand-caught by M.K.S. were captured in Bedford, MA, USA at the Concord Field Station in 2015. Table 2 lists the species of our six representative insect wings shown in Figs 2–5. The listed *Myrmeleontidae sp.* was collected by Dino J. Martins (Mpala Research Centre) in North Kajiado, Oloosirkon, Kenya in 2008.

**Table 1.**
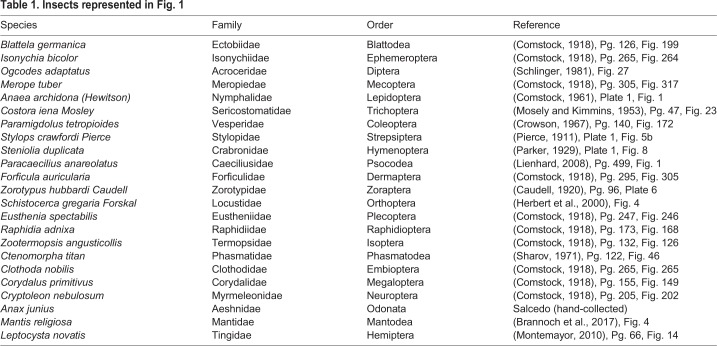
**Insects represented in** **Fig. 1**

**Table 2.**
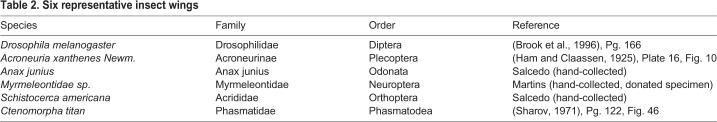
**Six representative insect wings**

### Image segmentation

To quantitatively characterize an insect wing, we first segmented wing images using a Level-Set approach from Hoffmann et al. (2018). For each polygonized wing, the coordinates were rescaled such that the entire wing had area 1. This effectively removed all size information for each wing, allowing comparison between species. In each of the analyses discussed, the area was first rescaled before applying the technique (Hoffmann et al., 2018). Due to the increased diversity of wing domain shapes, the procedure needed to be modified to handle an increased diversity of wing domains. Specifically, regions that were particularly non-convex needed to be properly accounted for. This was done through a combination of code changes (https://github.com/hoffmannjordan/size-and-shape-of-insect-wings and https://github.com/hoffmannjordan/insect-wing-venation-patterns) and by manually adding looped regions in certain parts of the wing. These looped regions served to help in non-convex wing domains. A loop will not polygonize out, but allowed us to add nodes when we polygonized that would not be otherwise captured.

This technique uses a preliminary thresholding algorithm to separate wing domains into connected components. A random point in each domain-component is chosen, from which a ‘front’ expands outward with a speed that is based on the background image intensity. Through veins, the front travels very slowly, while through open space, such as domains, the front travels rapidly. This algorithm tracks the fronts as a function of image intensity (thus differentiating between membrane and venated regions), and computes where multiple domains contact each other and tessellate the wing.

Using this polygonal reconstruction, we were able to accurately and efficiently compute many geometric properties of an insect wing that is only possible with well-segmented data. For each wing, we also used the connectivity of neighboring vein domains and vertices to construct an adjacency matrix describing topological relationships between neighboring vertices. We have discussed the geometric and topological properties as simple shape spaces, or morphospaces. These morphospaces separate wings using comparable geometric parameters, across species and orders. All codes are available online at https://github.com/hoffmannjordan/insect-wing-venation-patterns.

### Network analysis

We deployed a suite of network analysis tools on the diverse set of insect wing geometries. We tried both weighted (Morrison and Mahadevan, 2012) and unweighted analysis (Newman, 2006) (techniques are described below).

After polygonizing an insect wing, we construct a list of vertices. We then construct an adjacency matrix, *M*=*N*×*N* of 0’s, where *N* represents the number of vertices. For each *i*, *j* pair of vertices that are connected, we set *M*_*i*,*j*_=1.

Applying weighted connections between nodes (vein junctions) with length, *L*, allowed weighting *M*_*i*,*j*_=1/*L*^*n*^ where *L* is the distance between nodes *i* and *j* and *n*=1, 2. We also looked at an analysis using the resistance between nodes, *L*/*r*^4^, where *r* was computed using the 2D width from segmented images of original micrographs (giving us an approximate thickness of veins). Rather than measure the radius of each vein segment, we took a handful of original micrographs and measured approximately 50 cross vein radii and their location. We then interpolated over the wing as a proxy.

When computing curvature (Gander et al., 1994), *κ*, we did not use our polygonized wing. Instead, we used our original segmentation and extracted the boundary of the wing region. Then we oriented each wing such that the base of the wing was on the left and the perimeter of the wing ran clockwise. We chose a distance of *N*=0.02*L*_*P*_, and for each point on the perimeter, *P*, we took *p*_*i*_, *p*_*i*−*N*_ and *p*_*i*+*N*_ where *L* is length of the entire perimeter. From here, we performed a linear-least square fit to a circle, where we defined *κ*=1/*R*.

For all nodes *N*, we summed up the distances between all nodes. In doing this calculation, we then subtracted off the perimeter, which we calculated separately. In the corresponding main text figure, we omitted a selection of wings to improve visibility.

We characterized representatives of our large dataset using the P–D morphology traces introduced in (Hoffmann et al., 2018). We divided the wing into 25 equally spaced bins along the long axis of the insect wing. To calculate the mean circularity of each slice*i*, which we denoted 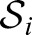, we computed(2)
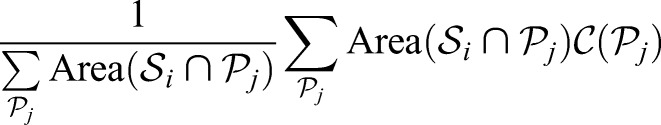
where the sum was applied over all polygons 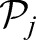. This produced a smoothly varying mean circularity as we moved along the long axis of the wing from base to wing tip. For polygon 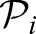, we obtained the area fraction *f*_*i*_ that overlapped with the bin. We constructed the vector of all area fractions in bin *i*, denoted 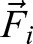. We also had the vector of all circularities 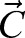 and all areas 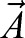. For each bin, we computed(3)
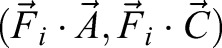
giving us the weighted mean area and the mean circularity of the wing domains in the region.

## Data Availability

Integer csv files of segmented images of all data along with code used in the manuscript are freely available. All codes and data can be found at: https://github.com/hoffmannjordan/size-and-shape-of-insect-wings. A more general segmentation code can be found at https://github.com/hoffmannjordan/Fast-Marching-Image-Segmentation.
